# The study of the *oipA* and *dupA* genes in *Helicobacter pylori* strains and their relationship with different gastroduodenal diseases 

**Published:** 2015

**Authors:** Negar Souod, Meysam Sarshar, Hossein Dabiri, Hassan Momtaz, Mohammad Kargar, Alireza Mohammadzadeh, Saeed Abdi

**Affiliations:** 1 *Young Researchers club, Central Tehran Branch, Islamic Azad University, Tehran, Iran*; 2 *Department of Public Health and Infectious Diseases, Sapienza, University of Rome, Rome, Italy *; 3 *Department of Clinical Microbiology, Faculty of Medicine, Shahid Beheshti University of Medical Sciences, Tehran, Iran *; 4 *Department of Microbiology, ShahreKord Branch, Islamic Azad University, ShahreKord, Iran*; 5 *Department of Microbiology, Jahrom Branch, Islamic Azad University, Jahrom, Iran*; 6 *Department of Microbiology, School of Medicine, Gonabad University of Medical Sciences, Gonabad, Iran*; 7 *Gastroenterology and Liver Diseases Research Centre, Research Institute for Gastroenterology and Liver Diseases, Shahid Beheshti University of Medical Sciences, Tehran Iran *

**Keywords:** *Helicobacter pylori*, *oipA*, *dupA*, *PCR*, *Gastric disorders*

## Abstract

**Aim:**

The purpose of this investigation was to determine the *oipA* and *dupA* genes of *Helicobacter pylori* isolates from west of Iran; Chaharmahalo Bakhtiyari region and find their relationship with the severity of the gastroduodenal diseases.

**Background:**

*Helicobacter pylori* is an organism responsible for many gastroduodenal diseases. Many studies suggest that genetic diversity in *H****. ****pylori* virulence factors such as *oipA* and *dupA* genes is high among isolates of different geographic regions and may cause more severe diseases.

**Patients and methods:**

In this cross-sectional study, gastric biopsy specimens were taken from 150 patients suffering from gastroduodenal diseases. The presence of *ureC*, *dupA *and* oipA *genes was tested by polymerase chain reaction (PCR).

**Results:**

Overall, 123 (82%) *H. pylori *strains were isolated from 150 specimens. *dupA* gene was detected in 41 (33.33%) *H.pylori*-positive specimens. There was a reverse correlation between this gene and gastric cancer. The *oipA* gene was found in 88 (71.54%) samples and statistically there was no association between this gene and gastric disorders. As statistical analyses revealed, the presence of the *dupA* was more common in isolates with the *oipA* negative.

**Conclusion:**

Based on our findings, the presence of *dupA* gene can be considered as a marker for the onset of severe diseases. However, the *oipA* gene cannot be regarded for prediction of gastroenterology diseases. Meanwhile, extended molecular epidemiology researches in other populations are recommended.

## Introduction

*Helicobacter pylori *is a major cause of chronic gastritis and involved in the pathogenesis of several diseases like gastric and duodenal ulcer, gastric adenocarcinoma, and mucosa-associated lymphoid tissue lymphoma (1-3). This bacterium has several virulence factors which are generally classified into three categories: The first group belongs to strain-specific genes, such as a *cag* pathogenicity island (PAI) and Plasticity Island genes (e.g. *jhp0947* and *dupA* genes) which do not exist in all *H. pylori *strains. The second group consists of phase-variable genes whose gene status can be changed during growth or under different conditions. Six genes encoding outer-membrane proteins (*babB*, *oipA*, *hopZ sabA*, *sabB* and *babC*) are thought to undergo phase variation. The last group consists of genes with variable genotypes or structures depending on the strain. For instance, specific *vacA *genotypes containing different mosaic combinations of signal regions (s), middle region (m) and intermediate region (i) allelic types have been associated with different clinical outcomes (4). Reports on the clinical predictive value of putative virulence factor status and disease outcomes are controversial based on different geographic regions (5-8). On the other hand, these factors are not independent and are often closely linked (e.g., the *cag *PAI, *vacA *s1, and the *babA2 *gene) making it difficult to classify which factor(s) has the most important predictor role in diseases severity and clinical manifestations (9). Several studies have provided new insights into the role of several putative virulence factors of H. pylori in gastroduodenal pathogenesis. Recently, the duodenal ulcer-promoting gene (dupA), which encompassed both *jhp0917* and* jhp0918* and located in the plasticity region of *H. pylori* genome, was identified (10). Interestingly, the *dupA* gene is homologous to virB4, a gene encoding a component protein of the type IV secretion system (TFSS) in *Agrobacterium tumefaciens* (3). The *jhp0917* and *jhp0918*, were examined by Lu et al. in 2005 and illustrated as a risk marker for prediction of duodenal ulcer disease and a protective factor against gastric cancer in strains isolated from Japan, Korea and Colombia (11). This continuous gene as a virulence marker was found to be more prevalent in patients with duodenal ulcer while was associated with a reduced risk for development of gastric atrophy and cancer in these populations (12, 13). In contrast, the *dupA* genotyping in some areas showed no association of this gene with duodenal ulcer, but suggested an association with gastric cancer (10, 14). 

The *H. pylori *outer inflammatory protein, OipA, is an important virulence factor which is associated with clinically important presentation of peptic ulcer, such as enhanced interleukin-8 secretion and increased inflammation (15). *H. pylori *contain either a functional or non-functional *oipA *gene. The functional status is regulated by the slipped strand repair mechanism based on the number of CT dinucleotide repeats in the 5ʹ region of the gene (9).

Considering the high prevalence of *H. pylori* infection in Iranian population, there are several reports about common virulence markers such as *vacA* and *cagA*, however there is very limited documents about *dupA* and *oipA*. The aim of our study was to assess the distribution of *dupA and oipA *genes in *H. pylori *strains isolated from patients with gastrointestinal disorders and to determine whether, these genes are associated with different clinical outcomes. 

## Patients and Methods

**Sample**

Sampling was performed over a year (March 2010 to February 2011) from patients with gastroduodenal diseases referred to endoscopy centre of Hagar hospital of Shahrekord city in the west of Iran. Prior to sampling the questionnaire including medical history and demographic data were recorded for each patient. Informed consent form, declaring their willingness for the application of their anonymous data for research purpose was obtained from all studied patients prior to endoscopy. The protocol was approved by the ethical committee of Shahrekord University of Medical Science. Four gastric punch biopsy specimens from antrum of the stomach of each patient were collected; two for histopathology study, one for RUT test and the other for PCR. 

**Rapid Urease Test (RUT)**

One piece of each specimen was examined by Rapid Urease Test (RUT) for detection of *H. pylori*. Rapid urease test was performed with a Gastro urease kit (Bahar-Afshan Co, Tehran, Iran) according to manufacturer’s instruction. 

*Preparation of genomic DNA and polymerase chain reaction: *A second piece from positive samples in RUT was used in PCR. DNA was extracted from biopsy specimens using a Genomic DNA purification kit (Qiagen, Hilden, Germany) according to manufacturer’s recommendations. The *16S rRNA* gene was amplified to confirm the presence of the isolated *H. pylori *strains. According to table 1, HP-1 and HP-2 primers designed and verified previously for this aim (16, 17). For analyses of the presence of target genes ( *dupA* and *oipA)*,* H.pylori* DNA was amplified using specific oligonucleotide primers (Table 1). Primers of *jhp0917 *and* jhp0918 *yielded fragments of approximately 307 and 276 bp, respectively. The primers of the *oipA* gene yielded a fragment of 401 bp. DNA samples from *H. pylori *(D0008; Genekam, Germany) were used as a positive control of *16S rRNA*, *dupA* and *oipA *genes, and sterile distilled water was used as a negative control. All PCR mixtures were prepared in a volume of 25 µL containing 1X PCR buffer, 0.4 µM of each primer, 0.3 U Taq DNA polymerase (CinnaGen Co., Tehran, Iran) and 300 ng DNA sample. The mixture placed in a thermocycler (Eppendrof Mastercycler 5330; Eppendorf-Nethel-Hinz GmbH, Hamburg, Germany), and PCR products were visualized by electrophoresis in 1.5% agarose gel, stained with ethidium bromide, and examined under ultraviolet illumination. 

**Table 1:** Primers used for PCR analysis of *ureC*, *dupA* and *oipA* genes

Gene	Primer	Primer sequence (5'-3')	Size (bp) of PCR product	Reference
*16S rRNA*	HP-1 HP-2	CTGGAGAGACTAAGCCCTCC ATTACTGACGCTGATTGTGC	109	16
*jhp0917 *(*virB4*)	JHP917 (+) JHP917 (_)	TGGTTTCTACTGACAGAGCGC AACACGCTGACAGGACAATCTCCC	307	11
*jhp0918 *(*virB4*)	JHP918 (+) JHP918 (_)	CCTATATCGCTAACGCGCGCTC AAGCTGAAGCGTTTGTAACG	276	11
*oipA*	HPO638F HPO638R	GTTTTTGATGCATGGGATTT GTGCATCTCTTATGGCTTT	401	23

**Data analysis**

 The data were analyzed using *SPSS* software (Version 17.*SPSS* Inc, USA) and *p *value was calculated using Chi-square and Fisher's exact tests to find any significant relationship. *P *values less than 0.05 were considered statistically significant.

**Table 2:** The frequency of the relationship between *dupA* and *oipA* genes and gastrointestinal diseases

Gene	Gastric Ulcer n=18	Duodenal Ulcer n=33	Gastric Cancer n=3	Gastritis n=120	Duodenit n=6
*dupA*	7(38.88%)	13(39.39%)	0(0.00%)	37(30.83)	2(33.33%)
*oipA*	14(77.77%)	27(81.81%)	1(33.33%)	88(73.33)	4(66.66%)

**Histopathology**

During endoscopy two biopsy specimens were taken from the antrum for histological evaluation. These specimens were fixed in 10% buffered formalin, embedded in paraffin, cut into sequential 4μm sections and stained with hematoxylin and eosin (H&E) and modified Giemsa stain. Multiple high-powered fields were examined by two pathologists blinded to the characteristics of *H.pylori* strains. 

## Results

150 patients with mean age of 46 ± 17 years, including 71 (47%) men and 79 (53%) women, were studied. Based on RUTs, 131(87%) of patients were *H.pylori* positive while according to PCR assays 123 (82%) specimens were confirmed to be *H.pylori* positive. The patients were classified at the time of endoscopy and histopathology as having gastritis ulcers (n=18), Duodenal ulcer (n=33), Gastric cancer (n=2), gastritis (n=120), and duodenitis (n=6). It should be noted that (63%) of patients had several diseases together. Individuals had some clinical symptoms such as pain (n=112), vomiting (n=43), inappetence (n=25), Acid reflux (n=41), and flatulence (n=39). Demographic factors such as gender, education, occupation and gastric drug usage, did not show any statistical correlation with *H. pylori* infection and clinical outcomes (P>0.05). But smoking (P<0.0001) and age (P= 0.02) were significantly associated with *H. pylori* infection. 

In this study, *dupA* gene was detected in 41 (33.33%) specimens. As table 2 shows, there was no significant relationship between *dupA* status and duodenal ulcer disease (P=0.25). However, there was a converse relationship between *dupA*- negative strains and gastric cancer disease (P=0.02). There was a considerable correlation between the presence of this gene and patient’s age (P=0.007), smoking (P=0.04) and individuals who suffer flatulence (P=0.03). The *oipA* genotype was detected in 88 (71.54%) of *H. pylori* positive samples. This gene was in relation with the age groups of patients (P=0.007) and was more common in patients with gastritis rather than other groups (P=0.001). There was a close relationship between infection with *oipA* positive *H. pylori* and the presence of vomiting (P=0.009) and stomach-ache (P=0.03) regardless of clinical outcomes. As statistical analyses revealed, the presence of the *dupA* were more common in isolates with the *oipA* negative (P<0.0001). 

## Discussion

*H. pylori *infection is very common worldwide. It is estimated that more than 50% of the world’s population are infected with* H. pylori *(14). The rapid changes in the epidemiology of different clinical outcomes caused by *H. pylori* suggest an interaction between an environmental factor, host and microbes that leads to a change in prevalence of strains differing in virulence (1, 6). Several studies have been evaluated the association between different virulence factors of *H. pylori* and clinical manifestations in Iranian population. There is limited information about *dupA* and *oipA * to define predictive value of virulence marker for gastric disorders. In the current survey, we have examined two *H. pylori* virulence factors; one is located in plasticity region (*dupA*) and the other is a phase variable gene (*oipA*). We also evaluated their relationship with different gastric disorders in the west of Iran.

The prevalence of *H. pylori *differs significantly both between and within countries, with high rates of infection being associated with low socioeconomic status and high densities of living (18, 19). For instance, in Japan, South America, Turkey and Pakistan, the prevalence is more than 80%, while in Scandinavia and England, the prevalence is between 20% and 40% (6, 7). The prevalence of this bacterium in Iran is 60-90%, indicating Iran is a high risk region for *H. pylori *infection. Douraghi et al and Dabiri et al, have studied these genes in Tehran, Iran (13, 15). However, no evaluation has been done in West of Iran. According to our results, the prevalence of this bacterium was 87% and 82% by RUT and PCR, respectively which is similar to previous reports from Iran (2, 15). Lu et al. demonstrated that the existence of the *jhp0917* and *jhp0918 *genes, which located in the plasticity region, forms one gene (*dupA*) (11). The correlation of the *dupA* with clinical outcome is still controversial. Some researchers have shown that the gene is associated with an increased risk of duodenal ulcer and protection against gastric atrophy, intestinal metaplasia and gastric carcinoma in Japan and Korea (3, 11). Likewise, the gene was shown to be protective against gastric carcinoma in Colombian patients (11). Quiroz et al. from Brazil and Imagawa et al. from Japan suggest that there is no association between *dupA* with duodenal diseases, but strains containing *dupA* without the stop codon polymorphisms were associated with lower risk for development of gastric carcinoma (20, 21). However, some groups such as Gomes et al. in Brazil and Nguyen et al. in Japan did not find any significant association between *dupA* gene and duodenal ulcer disease (14, 19). The prevalence of *dupA* (both *jhp0917* and *jhp918*) varies from 6% to 92% according to different studies around the world (3). Our results, similar to Lu et al. from Colombia and Zheng et al. from China showed that both *jhp0917* and *jhp0918* genes were present in 33.33% of isolates (11, 22). However, in our study, 6% of *H. pylori* strains did not contain *jhp0918* gene. This finding is similar to those of Archachi et al. that showed 11% of cases were negative for *jhp0918.* It is likely that *dupA* gene without *jhp0918* is not functional (12). When we came to analyze association of *dupA* status with clinical outcomes, our results were in accordance with Queiroza et al. and Imagawa et al. (20, 21). They showed there was no association between *dupA* gene and duodenal ulcer disease but there was a statistical significant association between the lack of this gene in strains and development of gastric cancer (20, 21). The presence of the *dupA* gene prevents the development of gastric cancer. The OipA is a member of the large outer membrane protein family whose functional status is regulated by slipped-strand mispairing based on the number of CT dinucleotide repeats in the 5ʹ region of the gene (a switch status of “on” indicates the gene is functional, and a switch status of “off” indicates it is non-functional) (15, 23). 

Using primers for detection of *oipA* gene, we figured out 71.54% of isolated strains contain this gene which is in accordance with our previous study that showed the *oipA* prevalence varies from 33% to 71% in Iranian population based on different ethnic background (13). In contrast with Yamaoka et al. and Kudo et al. identified the *oipA* gene from 45.9% and 30% of studied *H.pylori* isolates respectively (8, 24). In majority of studies, the *oipA* gene was present in most strains. In contrast, there were many *oipA*-negative cases in the current study. We used the same PCR primers as used in previous studies, which worked well both in Asian and Western strains. Therefore, there should be two possibilities: one is the nucleotide sequences of PCR primer regions are considerably different in Iranian strains from other countries and another possibility is that there are *oipA*-negative strains in Iran. More studies are needed for approving which possibilities will be applied to Iranian strains. Shao et al. declared there is no correlation between *oipA* gene and gastric diseases (25), while similar to our previous result; we interestingly found this gene to be significantly more common in non-ulcer dyspepsia patients rather than peptic ulcer dyspepsia cases (7, 15). Previously we have reported that the presence of the *oipA* is equal with low risk for GC development, while in the current study we did not find the same correlation (15). Overall, the presence of the *oipA* gene and clinical outcomes are still unclear. In previous studies, the *oipA* gene was present in most strains and the *oipA* status was evaluated by functional status (i.e. ‘on’ or ‘off’ status). As the numbers of patients in the current study were relatively small, further studies with larger numbers are necessary to clarify the roles of *oipA* in clinical outcomes. As a result of our findings, there was a statistically significant relationship between the lack of *oipA* gene and the presence of *dupA* in isolated strains. This is compatible with those of Matteo et al., which suggested that these genes are present with each other only in one tenth of strains (5). 

In conclusion, according to the results there was a reverse correlation between the *dupA* gene presence and gastric cancer as well as *dupA* and *oipA* gene. While the *oipA* gene is only statistically associated with gastritis, which is not consider as a severe *dupA* gene, an important marker for more severe gastrointestinal disease prediction. However, this fact does not apply for *oipA* gene among patients in the west of Iran. Finally, further and extended molecular epidemiology researches in other parts of Iran are recommended.
